# Complete mitochondrial genome of Triplophysa nasobarbatula

**DOI:** 10.1080/23802359.2020.1745099

**Published:** 2020-11-23

**Authors:** Xu Yang, Huamei Wen, Tao Luo, Jiang Zhou

**Affiliations:** a Guizhou Normal University, Guiyang, China;; b Central China Normal University, Wuhan, China

**Keywords:** Mitochondrial genome, phylogenetic tree, *Triplophysa nasobarbatula*

## Abstract

The complete mitochondrial DNA genome of *Triplophysa nasobarbatula* was sequenced and characterized. *Triplophysa nasobarbatula* revealed that the complete length of its mitochondrial genome was 16,316 bp, composed of A (29.71%), C (24.79%), G (17.22%), T (28.29%), A + T (57.99%), and C + G (42.01%). Its genetic constitution and arrangement were consistent with the taxon of the Teleost, including 13 protein-coding genes, 22 tRNA genes, 2 rRNA genes, and 2 main non-coding regions, D-loop region and O_L_ region. All genes were encoded by the H-strand, except for 1 protein-coding gene (ND6) and 8 tRNA genes (tRNA-Gln, tRNA-Ala, tRNA-Cys, tRNA-Asn, tRNA-Tyr, tRNA-Ser, tRNA-Glu and tRNA-Pro) are encoded by the L-strand. Our mitochondrial genome data may provide information for taxonomic resolution, taxonomic resolution, and other studies about this genus of *Triplophysa*.

The *Triplophysa nasobarbatula* belongs to the family Nemacheilidae, which is widely distributed in the rivers and lakes of QinghaiTibet Plateau and adjacent ranges (Zhu 1989; Du et al. 2008). In China, the cave-dwelling species of *Triplophysa* occur in the Karst area of Yunnan Province, Chongqing City, Hunnan Province, Guangxi Zhuang Autonmous Region, and Guizhou Province. Balitoridae and their phylogenetic analyses are very important for studying the environmental adaptability of freshwater fishes (Doadrio and Perdices 2005; Perdices et al. 2012). In this study, we determined the mitochondrial genome (16,316 bp; GenBank accession no. MH685911.1) of *T. nasobarbatula*, hoping to enrich mtDNA data of the genus. The specimen was collected from a unnamed stream in Liujiang River (25°28′57.09″N, 108°06′23.17″E), Libo county, Guizhou Province of China in January 2019. It was stored in the animal specimen room of the School of Karst Sciences (GZNU20190114001), Guizhou Normal University, Guiyang, China.

Total DNA was extracted from the fish muscle tissues and used the second-generation high-throughput sequence to measure the complete mitochondrial genome. The mitochondrial genome is identical to that found in most teleost mitochondrial genomes. The entire mitochondrial genome consists of 13 protein-coding genes, 22 tRNA genes, 2 rRNA genes, and two main non-coding regions, D-loop region and O_L_ region. All genes were encoded by the heavy strand H-strand, except for 1 protein-coding gene (ND6) and 8 tRNA genes (tRNA-Gln, tRNA-Ala, tRNA-Cys, tRNA-Asn, tRNA-Tyr, tRNA-Ser, tRNA-Glu and tRNA-Pro), which were encoded by the L-strand (Boore 1999). The overall base composition of the *T. nasobarbatula* mitochondrial genome was A (29.71%), C (24.79%), G (17.22%), T (28.29%), A + T (57.99%) and C + G (42.01%), which is an A + T-rich pattern of the vertebrate mitochondrial genomes (Mayfield and McKenna 1978). The total length of the 13 protein-coding genes was 11,385 bp in length, the longest one was ND5 (1809 bp) and the shortest was ATP8 (162 bp), Cytb has 1131 bp. The 12S rRNA was 947 bp long and the 16S rRNA was 1638 bp in length. All the protein-coding genes initiated with the ATG start codon except for CO1.

Complete mitochondrial DNA sequences of 11 species of *Triplophysa* and 1 species of *Cobitis striata* were obtained from GenBank, and *C. striata* was set as an outer group. The phylogenetic tree was established using the maximum likelihood (ML) method that was conducted in IQ-TREE (Nguyen et al. 2015) with ultrafast 100 bootstrapping (Hoang et al. 2018).

The maximum-likelihood (ML) phylogenetic tree established using complete mitochondrial DNA sequences at the genus level is shown in Figure 1. The results showed that *Triplophysa* were divided into three branches, while *T. nasobarbatula* and typical cave-dwelling *Triplophysa* species gather into a branch. According to the existing research, all the species on this branch discovered in the karst area of South China.

**Figure 1.**
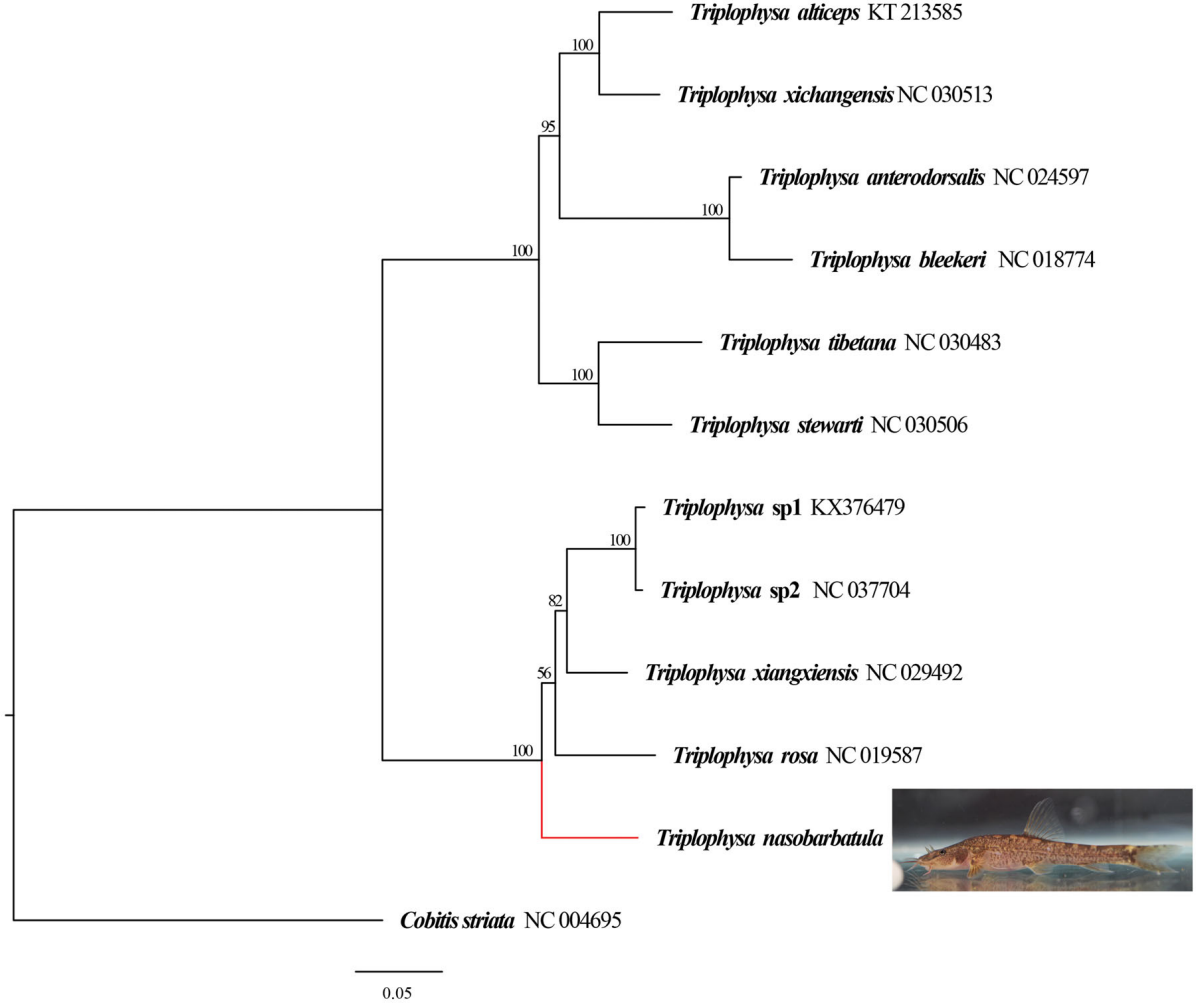
ML phylogenetic tree based on the complete mitochondrial genome. ML bootstrap values are shown above nodes. The mitochondrial genome sequences of analyzed species were obtained from the GenBank databases.
